# Caseous calcification of the mitral annulus (CCMA) – A rare mimicker of a cardiac mass

**DOI:** 10.1016/j.radcr.2022.04.042

**Published:** 2022-05-29

**Authors:** Nirmal Prasad Neupane, Kritisha Rajlawot, Chandramani Adhikari, Dipanker Prajapati

**Affiliations:** a Radiologist, Department of Radiology, Shahid Gangalal National Heart Centre, Kathmandu, Nepal; b Cardiologist, Department of Cardiology, Shahid Gangalal National Heart Centre, Kathmandu, Nepal

**Keywords:** Caseous calcification of mitral annulus, Cardiac MRI, Cardiac CT

## Abstract

Caseous calcification of the mitral annulus (CCMA) is a less common variant of mitral annulus calcification mimicking an intracardiac mass. The caseous calcification of the mitral annulus is a harmless benign entity that is an infrequent and incidental finding. Awareness about the condition is a must to avoid misinterpretations leading to unnecessary investigations and interventions. Therefore, here we present a case of CCMA diagnosed through different radiological modalities such as cardiac magnetic resonance imaging and multidetector computed tomography.

## Introduction

Caseous calcification of the mitral annulus (CCMA) is a less common variant of mitral annulus calcification mimicking an intracardiac mass. It is a result of a prolonged degenerative process in the mitral valve fibrous ring where the posterior fibrous mitral annulus is commonly affected [1]. Being a rare form of variant, it is often a challenge for clinicians to diagnose a CCMA only based on an echocardiogram. Therefore, here we present a case of CCMA diagnosed through different radiological modalities such as cardiac magnetic resonance imaging (CMR) and multidetector computed tomography (MDCT).

## Case history

A 65 years old female patient presented to our Out-Patient Department for a routine checkup. The only medical history she had were hypertension and dyslipidemia. She underwent a routine echocardiogram after which she was sent for a CMR with the suspicion of an intracardiac mass in the left atrium.

## Imaging findings

**Fig. 1:**
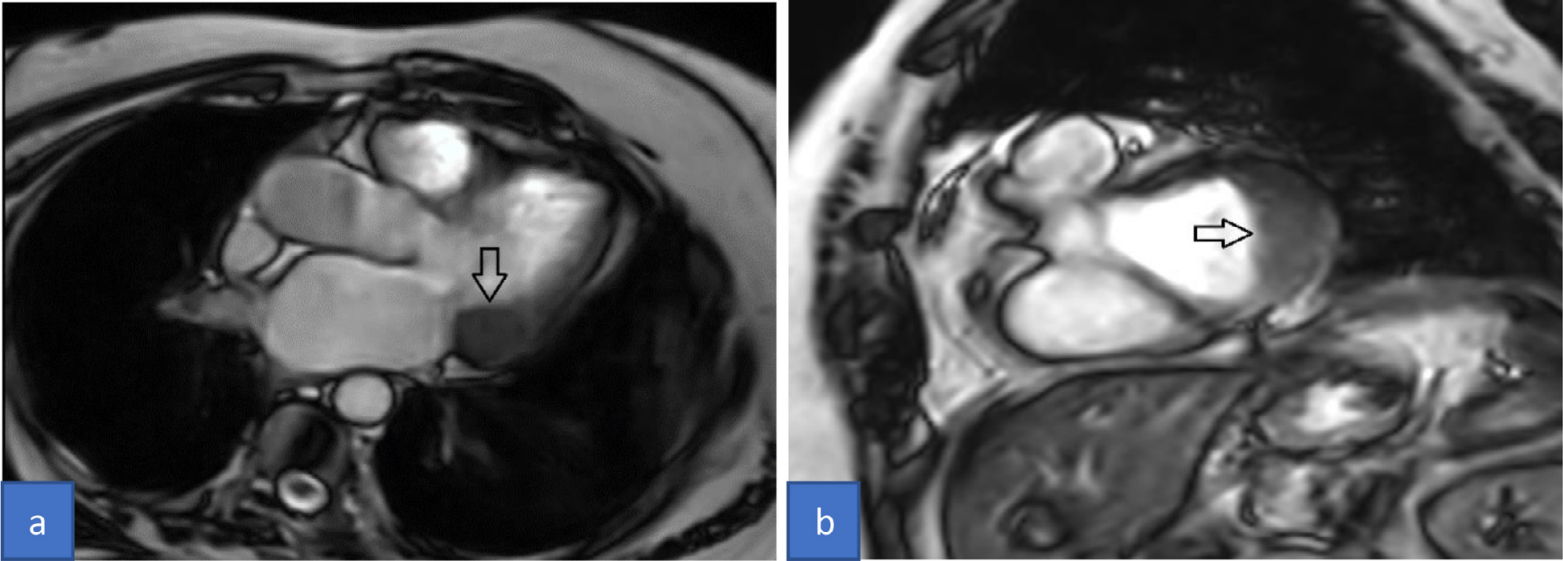
Cardiac Magnetic Resonance (CMR) cine images 3- chamber view (A) and short-axis view (B) showing iso to low signal intensity lesion posteroinferior to the posterior leaflet of the mitral valve (arrow).

**Fig. 2:**
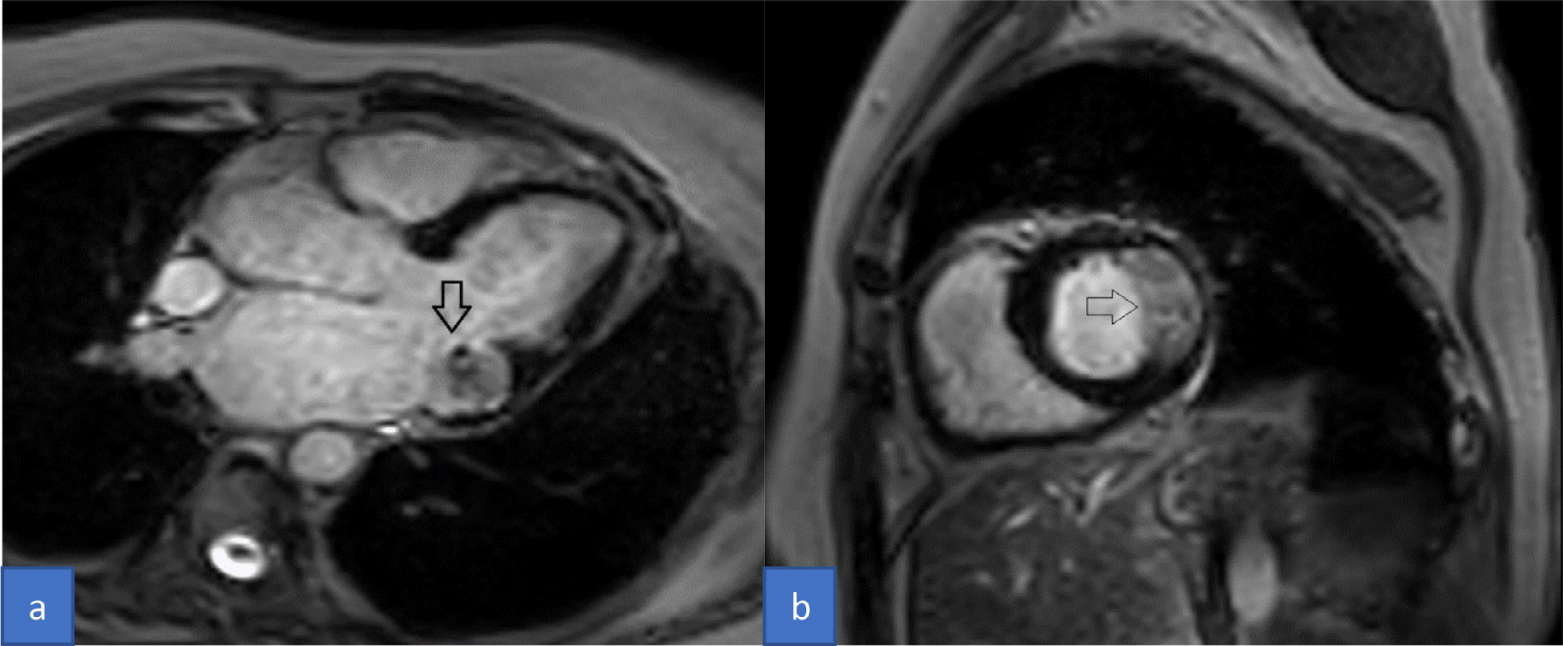
Cardiac Magnetic Resonance (CMR) delayed gadolinium contrast images 3-chamber view (A) and short-axis view (B) showing delayed enhancing peripheral rim with patchy enhancement in the center (arrow).

**Fig. 3:**
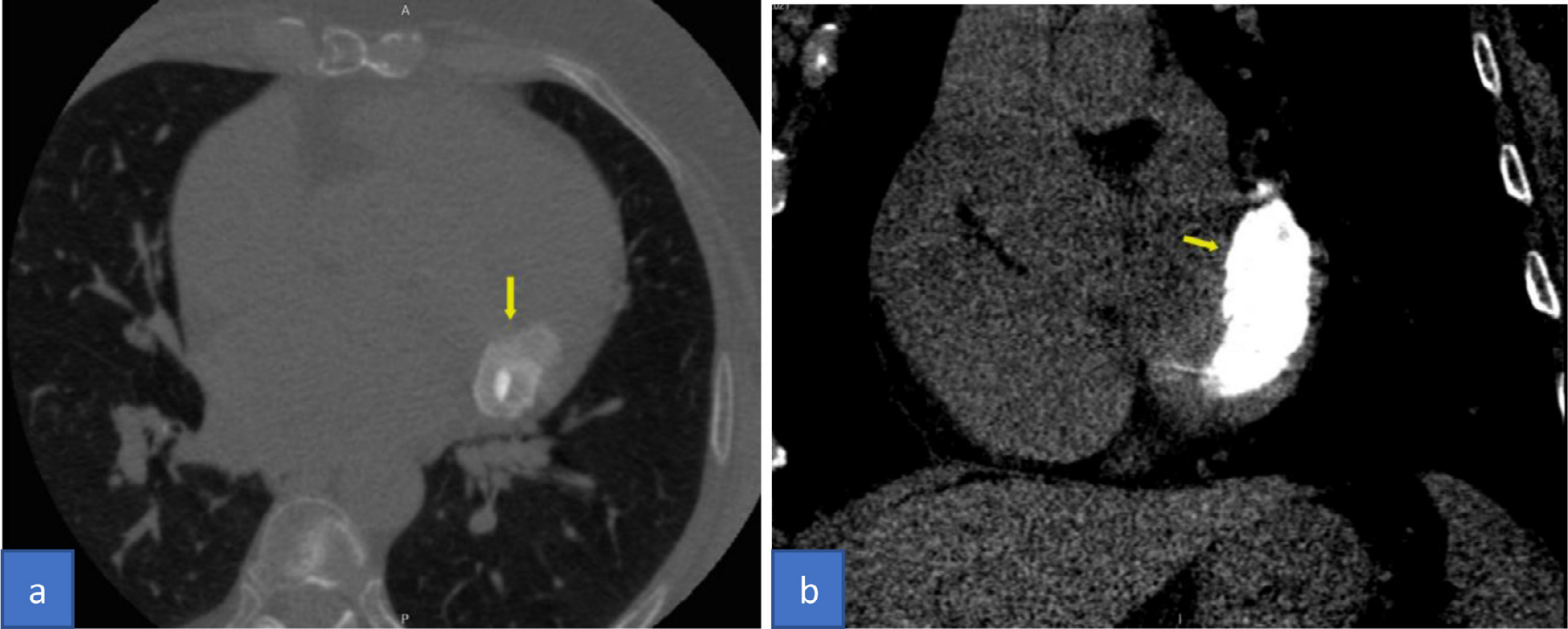
Computed tomography (CT) chest without contrast Axial view lung window (A) and coronal view soft tissue window (B) shows diffuse calcification of the mitral annulus mimicking a hyperdense mass (arrow).

Cardiac MRI with contrast was performed for the patient on a 3Tesla platform. Her CMR findings showed an approximately 3.4 × 1.8 × 1.8 cm sized T1 and T2 iso to low signal intensity lesion in the posteroinferior aspect of the posterior leaflet of the mitral valve (Fig. 1). The lesion extended and involved the posterior annulus and the posterior mitral leaflet. There was no evidence of prolapse of the lesion through the valvular orifice. The first pass gadolinium contrast enhancement study showed no enhancement of the lesion. However, on delayed GAD images, the lesion showed enhancing peripheral rim and central patchy enhancement (Fig. 2). All cardiac chambers were normal except for a dilated left atrium. With the typical location and characteristics of the lesion, we suspected CCMA and took the patient for a non-contrast cardiac CT. Hence, through a CT correlation, a diffuse calcification of the mitral annulus was confirmed giving a final diagnosis of the Caseous Calcification of the Mitral Annulus (Fig. 3).

## Discussion

CCMA is a rare but one of the various differentials for mass-like lesions in mitral valve including tumors such as myxoma, abscesses, vegetations, and thrombus [1]. As this variant of mitral annulus calcification does not have specific clinical symptoms, oftentimes it might get overlooked on routine clinical examinations. However, it is usually seen in elderly women with a history of high blood pressure, which was the comorbidity present in our patient as well. Diagnosis of CCMA is usually based on multimodality imaging approach. On CMR, the characteristic appearance of CCMA shows a low signal intensity mass in T1 and T2 weighted sequences, suggesting the calcium content. In a post-contrast study, late gadolinium enhancement sequences may depict a peripheral rim enhancement and show a lack of enhancement of the entire mass [1,2]. Furthermore, a well-defined hyperdense lesion with high HU values associated with diffuse calcification and no contrast enhancement are the typical findings of CCMA on cardiac CT [1,3]. All the other simulations of intracardiac mass however may be associated with specific clinical symptoms, with or without significant calcification or show enhancement in post-contrast studies. In summary, the primary imaging features of CCMA include posterior mitral valve annulus involvement, the presence of calcifications on CT, and a lack of contrast enhancement on MRI.

## Conclusion

The caseous calcification of the mitral annulus is a harmless benign entity that is an infrequent and incidental finding. The radiologic modality such as MDCT may facilitate to encounter this accidental lesion more regularly in clinical practice. At the same time, cardiac MR is another advanced modality that helps to rule out other differentials for intracardiac mass. Hence, awareness about the condition is a must to avoid misinterpretations leading to unnecessary investigations and interventions.

## Patient consent

Written informed consent has been taken from the patient for the publication of the case report.

## References

[1] Pradella S, Verna S, Addeo G, Oddo A, Miele V. (2019). Caseous calcification of the mitral annulus. J Radiol Case Rep.

[2] Hamdi I, Chourabi C, Arous Y, Ghommidh M, Houissa K, Haggui A (2018). Multimodality imaging assessment of a caseous calcification of the mitral valve annulus. J Saudi Heart Assoc.

[3] Gulati A, Chan C, Duncan A, Raza S, Kilner PJ, Pepper J. (2011). Multimodality cardiac imaging in the evaluation of mitral annular caseous calcification. Circulation.

